# Superior semicircular canal dehiscence postoperative outcomes: a case series of 350 repairs

**DOI:** 10.1007/s00701-024-06115-w

**Published:** 2024-05-24

**Authors:** Mahlet Mekonnen, Meachelle Lum, Courtney Duong, Shivam Rana, Khashayar Mozaffari, Gabrielle E. A. Hovis, Isaac Yang

**Affiliations:** 1 Department of Neurosurgery, University of California, Los Angeles, 300 Stein Plaza, Suite 562, Los Angeles, CA 90095-1761 USA; 2 Radiation Oncology, Los Angeles (UCLA), Los Angeles, CA USA; 3 Head and Neck Surgery, Los Angeles (UCLA), Los Angeles, CA USA; 4 Jonsson Comprehensive Cancer Center, Los Angeles (UCLA), Los Angeles, CA USA; 5 Los Angeles Biomedical Research Institute, Los Angeles (UCLA), Los Angeles, CA USA; 6 Harbor-UCLA Medical Center, Los Angeles (UCLA), Los Angeles, CA USA; 7 David Geffen School of Medicine, Los Angeles (UCLA), Los Angeles, CA USA

**Keywords:** Middle cranial fossa, Superior semicircular canal dehiscence, Case series, Postoperative outcomes

## Abstract

**Background:**

Superior Semicircular Canal Dehiscence (SSCD) is a dehiscence of the otic capsule which normally lies over the superior semicircular canal. This database constitutes the largest series of SSCD patients to date.

**Objective:**

To determine what preoperative factors, if any, contribute to postoperative outcomes and evaluate symptom resolution in a large SSCD patient cohort.

**Methods:**

A single-institution, retrospective chart review collected patient demographics, intraoperative findings, and pre-and postoperative symptoms. Fisher's exact t-test was performed for unpaired categorical variables, with a significance level of *p* < 0.05.

**Results:**

350 SSCD repairs were performed. The median age was 52 years (range: 17—86 years, ± 6.4 years), and the median follow-up duration was 4.6 months (range: 0.03—59.5 months, ± 6.8 months). Preoperative hearing loss was significantly associated with female sex (*p* = 0.0028). The most reported preoperative symptoms were tinnitus (77.4%), dizziness (74.0%), autophony (66.3%), amplification (63.7%), and disequilibrium (62.6%). Between patients who received unilateral versus bilateral SSCD repair, the greatest postoperative symptomatic resolution was seen in autophony (74.9%, *p* < 0.001), amplification (77.3%, *p* = 0.00027), hyperacusis (77.4%, *p* = 0.023), hearing (62.9%, *p* = 0.0063), and dizziness (54.6%, *p* < 0.001) for patients with unilateral SSCD repair.

**Conclusion:**

Surgical repair via the middle cranial fossa approach can significantly resolve auditory, vestibular, and neurological symptoms of patients with SSCD. Although this is one of the largest single-institution SSCD studies to date, future multi-institutional, prospective studies would be beneficial to validate these results.

## Introduction

Since the initial description by Minor et al*.,* 1998, superior semicircular canal dehiscence (SSCD) has been increasingly recognized and explored by neurosurgeons as a rare syndrome of debilitating vestibular and auditory symptoms resulting from a dehiscence in the bone overlying the superior semicircular canal [4, 19, 20, 36]. In SSCD, thinning or dehiscing of the petrous temporal bone superior to the superior semicircular canal (SSC) creates an open “third mobile window.” [4, 29, 37] Atypical communication of the SSC with the middle cranial fossa (MCF) and disturbed endolymph dynamics contribute to the auditory, vestibular, and neurological symptoms associated with SSCD [4, 15, 19, 26, 28, 29, 34, 36, 37]. High resolution computed tomography (HRCT) and cervical or ocular Vestibular Evoked Myogenic Potentials (cVEMP and oVEMP, respectively) are used to confirm SSCD diagnosis [4, 26, 34, 37]. Patients with SSCD can have impaired auditory perception, balance, and various other neurological deficits due to the altered otic capsule housing the vestibulocochlear structure. [36]

SSCD patients may present with auditory symptoms such as pulsatile tinnitus or tinnitus aurium, hearing loss, aural fullness, autophony, hyperacusis, internal amplification of visceral or eye sounds, or vestibular symptoms including vertigo, dizziness, balance disequilibrium, and oscillopsia among others [4, 12, 15, 26, 34, 37]. Tullio phenomenon and Hennebert signs, in which sound and pressure changes induce vertigo, are also observed in SSCD patients [4, 26, 37]. Current literature suggests that 33—63% of patients present with bilateral SSCD [15, 26, 34]. While less debilitating symptoms are managed conservatively,[4, 5, 15, 36] debilitating symptoms often make patients surgical candidates [4, 15]. Thin bone, or thinning instead of complete dehiscence of the bone overlaying the SSC, can also lead to the onset of vestibulocochlear symptoms [3, 33]. Interestingly, previous studies have found no association between thin bone and age or gender, as it has been hypothesized that these two factors could contribute to osteoporosis leading to SSCD [13, 23]. Systematic analysis has also shown age and gender to not be predictors of symptom outcomes postoperatively [23]. SSCD, however, has been correlated to bone dehiscence in other locations. [2]

Since its discovery, SSCD surgical approaches have evolved to be less invasive. Techniques have yet to be standardized [6, 18, 22, 25, 28, 29, 36]. Surgical materials are also unstandardized, varying from bone-wax, cortical bone chip, muscle, and others [28, 29]. Resurfacing, or covering the dehiscence, and plugging, or filling the entire dehiscence canal, are the two treatment techniques used. There is no consensus for which is better [29]. Common approaches of surgical repairs include the transmastoid and MCF approach [27]. The transmastoid approach requires drilling through the mastoid bone to access the dehiscence, and previous studies have suggested lower infection rates are associated with recovery [9, 11]. The MCF approach accesses the dehiscence from above, providing more direct exposure and greater symptom resolution compared to transmastoid approach [17, 22]. Along with surgical approaches and treatment, previous studies have also investigated symptom resolution and preoperative factors contributing to postoperative outcomes. [7, 12, 21, 22, 28, 30]

In the current study, we evaluate 350 SSCD surgeries performed over the last decade by the same neurosurgeon and neuro-otologist pair via the MCF approach. This database constitutes the largest SSCD database to date, as we attempt to delineate symptom resolution based on varying demographic factors.

## Methods

### Study population

**Fig. 1:**
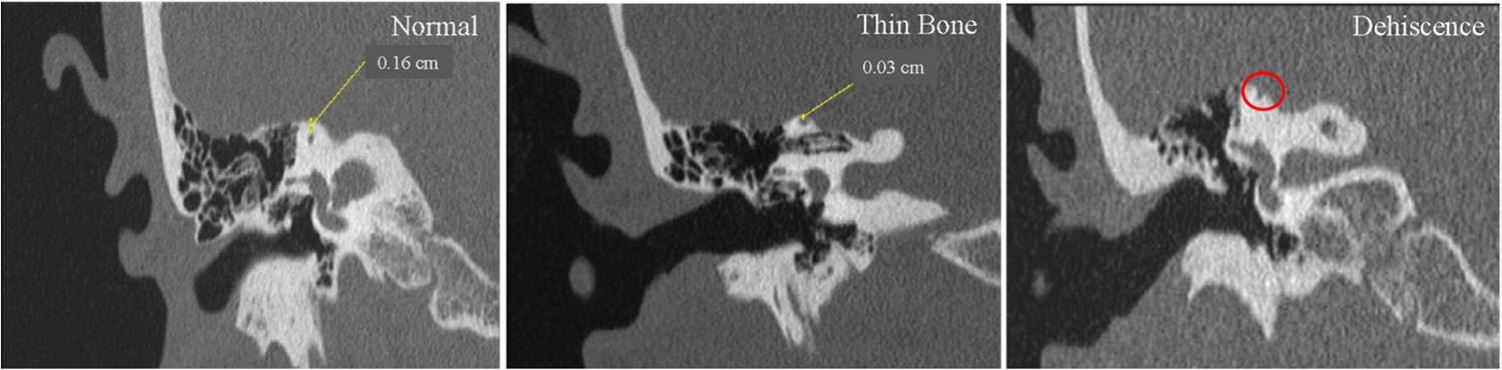
High-resolution coronal CT scans of the temporal bone illustrating the distance between the roof of the SSC and the cranial cavity

This study was approved by our institutional review board and a complete waiver of consent was obtained (IRB# 21–001139). Retrospective analysis was performed on 279 SSCD patients who underwent 350 surgical repairs via the MCF approach performed by the senior authors. Individuals who underwent SSCD repair between March 2011 and April 2021 that met inclusion criteria: (1) at least one characteristic of audiological and vestibular symptoms, (2) CT indicating absent bone over the SSC, and (3) at least one objective evidence of third window physiology were included. Diagnostic evaluation incorporated clinical presentation, physiologic testing, and HRCT, aligning with Bárány society recommendations [31]. Patients were divided into two unique cohorts according to laterality of tumor and operation. Cohort A consisted of patients with unilateral or bilateral SSCD who underwent a single, unilateral repair. Cohort B consisted of patients with bilateral SSCD who underwent bilateral repair consisting of two operations. Perioperative data for Cohort B was recorded for the second-side surgery. Revision was defined as repeat surgery on the ipsilateral side. HRCT scans of the temporal bones with 0.6 mm thickness were assessed to determine whether the patient had dehiscence or thinning of the bone covering the SSC. The bone measurement was the shortest perpendicular distance between the roof of the SSC and the cranial cavity (Fig. 1). The senior authors differentiated between a true dehiscence and thin bone using a bone thickness of > 0.5 mm to define a true dehiscence.

### Surgical approach

All SSCD repairs were performed via the MCF approach. The dehiscence was identified through use of microscopic visualization and intraoperative neuronavigational technology involving MRI and CT fusion images. The keyhole craniectomy method used was a minimally invasive craniectomy roughly 1.7 cm in diameter allowing for more direct access to the dehiscence. During the procedure, efforts were made to repair the defect without occluding the canal. Bone-wax, cortical bone chip, muscle, and other materials were used to resurface or plug the dehiscence as previously described. [2, 4, 12, 15, 21–23, 28, 34, 36]

### Data collection

Patient history and demographic information was collected from electronic medical records. This information included gender, age, SSCD laterality/surgical side, history of head trauma, perioperative cerebrospinal fluid (CSF) leak, surgical revisions, presence of contralateral thin bone, ear anomalies such as mastoiditis and Meniere's disease, and previous ear surgeries such as stapedectomies, round window occlusion, and tympanostomy tube placement. Autophony, amplification, aural fullness, tinnitus, hyperacusis, hearing loss, vertigo, dizziness, disequilibrium, oscillopsia, and headache were coded if clearly stated in the consultation and progress notes for preoperative and postoperative symptoms. Postoperative symptoms were recorded only if there was a specific mention in the most recent progress and follow up notes. Intraoperative findings were extracted from operative notes. For patients with bilateral SSCD that underwent bilateral treatment, pre- and postoperative symptoms were extracted from the consult, progress, and follow up notes associated with each specific side’s surgical repair. Similarly, pre-and postoperative symptoms of unilateral SSCD patients who had ipsilateral revisional surgery were extracted separately from the first surgery.

### Statistical analysis

Demographics along with medical and intraoperative risk factors and symptoms were analyzed through binary statistical variables. Univariate and multivariate analyses were performed to assess the relationships among patient demographics, preoperative symptoms, intraoperative findings, and postoperative outcomes. Chi-square test or Fischer’s exact test were used for comparison of categorical variables and Student’s t-test or Wilcoxon’s rank sum test were used for continuous variables. Fischer’s exact test was used for comparison of postoperative symptom resolution between patients with unilateral and bilateral SSCD repair, with a 0.05 $\alpha$ level. All statistical analyses were performed using R (version 3.4.3, The R Foundation for Statistical Computing, Vienna, Austria).

## Results

### Patient demographics and characteristics

A total of 350 SSCD repairs were performed among 279 SSCD patients between 2011 and 2021. 62.9% of the cohort was female. The median age was 52 years (range: 17—86 years, ± 6.4 years), and the median follow-up duration was 4.6 months (range: 0.03—59.5 months, ± 6.8 months). Patients underwent unilateral surgery (Cohort A) in 73.1% and bilateral repair (Cohort B) in 26.9%. Unilateral SSCD was seen in 46% of the cases and bilateral SSCD in 54%. Of the patients with bilateral SSCD, 57.7% underwent solely unilateral repair (Cohort A) and did not elect for later contralateral surgery. Revisional surgery was performed in 6.3%. All cases were repaired via the MCF approach. History of ear anomaly (mastoiditis, Meniere's disease, and benign paroxysmal positional vertigo, etc.) was recorded in 22.9% of patients. History of ear trauma (auricular hematoma, tympanic membrane perforation, and temporal bone fracture) was seen in 17.8% of cases. Perioperative CSF leak was seen in 25.4% of the cohort. Patient demographics and intraoperative findings are shown in Table 1**.** Chi-square test revealed a significant association between female sex and the presence of preoperative hearing loss, *p* = 0.0028. The most reported preoperative symptoms were tinnitus (77.4%), dizziness (74.0%), autophony (66.3%), amplification (63.7%), disequilibrium (62.6%), and aural fullness (50.6%) (Table 2).

**Table 1:** Patient demographics and intraoperative findings

Subgroup Analysis				
Characteristics	All Cases (N = 350)	^†^Cohort A (N = 256)	^‡^ Cohort B (N = 94)	^§^Revision (N = 22)
Age. yrs	52 ± 6.4	51 ± 6.2	51 ± 6.3	50.5 ± 5.9
Sex Male, N (%) Female, N (%)	130 (37.1) 220 (62.9)	105 (41.1) 151 (58.9)	25 (26.6) 69 (73.4)	8 (36.4) 14 (63.6)
Unilateral, N (%)	161 (46.0)	146 (57.0)	15 (15.9)	15 (68.2)
Bilateral, N (%)	189 (54.0)	110 (42.9)	79 (84.0)	7 (31.8)
Trauma, N (%)	62 (17.8)	42 (16.4)	20 (21.3)	4 (18.2)
Contralateral Thin Bone, N (%)	52 (14.9)	43 (16.8)	9 (9.6)	2 (9.1)
Surgical Side Left, N (%) Right, N (%)	195 (55.7) 155 (44.3)	149 (58.2) 107 (41.8)	46 (48.9) 48 (51.1)	14 (63.6) 8 (36.4)
Surgery Duration (Mean, hrs.)	2.45	3.1	1.8	1.9
CSF Leak, N (%)	89 (25.4)	60 (23.4)	29 (30.9)	11(50.0)
EBL (Mean, cc)	29.1	26.8	31.4	31.1
Follow up Mean (Mo.)	4.6 ± 6.8	4.5 ± 6.6	4.4 ± 6.7	4.2 ± 6.4

CSF: Cerebrospinal Fluid; EBL: Estimated Blood Loss

^†^ SSCD cases with unilateral SSCD repair

^‡^ SSCD cases with bilateral SSCD repair

^§^ SSCD cases with repeat repair on the ipsilateral ear, including patients from both Cohorts A and B

**Table 2:** Preoperative symptoms in patients who received unilateral, bilateral, and revisional repairs

Subgroup Analysis				
Characteristics	All Cases (N = 350)	^†^Cohort A (N = 256)	^‡^ Cohort B (N = 94)	^§^Revision (N = 22)
Autophony, N (%)	232 (66.3)	183 (71.5)	49 (52.1)	12 (54.5)
Amplification, N (%)	223 (63.7)	172 (67.2)	51 (54.3)	10 (45.5)
Aural fullness, N (%)	177 (50.6)	131 (51.2)	46 (48.9)	8 (36.4)
Tinnitus, N (%)	271 (77.4)	204 (79.7)	67 (71.3)	17 (77.3)
Hyperacusis, N (%)	160 (45.7)	124 (48.4)	36 (38.3)	10 (45.5)
Hearing loss, N (%)	170 (48.6)	124 (48.4)	46 (48.9)	7 (31.8)
Vertigo, N (%)	105 (30.0)	75 (29.3)	30 (31.9)	7 (31.8)
Dizziness, N (%)	259 (74.0)	196 (76.6)	63 (67.0)	9 (40.9)
Disequilibrium, N (%)	219 (62.6)	153 (59.8)	66 (70.2)	13 (59.1)
Oscillopsia, N (%)	94 (26.9)	72 (28.1)	22 (23.4)	5 (22.7)
Headache, N (%)	109 (31.1)	80 (31.3)	29 (30.9)	4 (18.2)

^†^ SSCD cases with unilateral SSCD repair

^‡^ SSCD cases with bilateral SSCD repair

^§^ SSCD cases with repeat repair on the ipsilateral ear, including patients from both Cohorts A and B

### Postoperative symptomatic resolution

Unilateral SSCD cases showed greater postoperative symptomatic resolution of dizziness compared to bilateral cases, *p* = 0.0023. When comparing postoperative symptomatic resolution between unilateral (Cohort A) and bilateral (Cohort B) SSCD repair cases, statistically significant postoperative improvement was seen in autophony (74.9%, *p* < 0.001), amplification (77.3%, *p* = 0.00027), hyperacusis (77.4%, *p* = 0.023), hearing (62.9%, *p* = 0.0063), and dizziness (54.6%, *p* < 0.001) in those with unilateral SSCD repair cases (Table 3). Patients in Cohort B showed the greatest postoperative resolution of vertigo (70.0%), but no significance was found in comparison to Cohort A. 85.7%, 80.0%, 75.0%, and 70.0% of patients with revisional surgery showed resolution of postoperative hearing, oscillopsia, autophony, and amplification, respectively. Multivariate analysis evaluated preoperative risk factors and postoperative symptoms adjusting for trauma and CSF leaks as potential confounders. No significant associations were found, *p* = 1.

**Table 3:** Postoperative symptomatic resolution with respect to laterality of SSCD repair

Subgroup Analysis				
Postoperative Symptom Resolution^**^	All Cases	†Cohort A	‡ Cohort B	P-value
Autophony, N (%)	164 (70.7)	137 (74.9)	27 (55.1)	* < .001
Amplification, N (%)	163 (73.1)	133 (77.3)	30 (58.8)	*.00027
Aural fullness, N (%)	93 (52.5)	73 (55.7)	20 (43.5)	.22
Tinnitus, N (%)	142 (52.4)	110 (53.9)	32 (47.8)	.14
Hyperacusis, N (%)	118 (73.8)	96 (77.4)	22 (61.1)	*.023
Hearing, N (%)	96 (56.5)	78 (62.9)	18 (39.1)	*.0063
Vertigo, N (%)	78 (74.3)	57 (76.0)	21 (70.0)	.93
Dizziness, N (%)	125 (48.3)	107 (54.6)	18 (28.6)	* < .001
Disequilibrium, N (%)	116 (52.9)	90 (58.8)	26 (39.4)	.19
Oscillopsia, N (%)	60 (63.8)	49 (68.1)	11 (50.0)	.061
Headache, N (%)	54 (49.5)	46 (57.5)	8 (27.6)	.083

^*^ Indicates statistical significance

^**^ All percentages are reported in respect to the presence of the symptom preoperatively, rather than the number of patients in each cohort

^†^ SSCD cases with unilateral SSCD repair

^‡^ SSCD cases with bilateral SSCD repair

^§^ SSCD cases with revisional repair on the ipsilateral ear

## Discussion

SSCD is a rare auditory and vestibular disorder in which there is a bone dehiscence located in the SSC of the inner ear. In normal patients without dehiscence, the inner ear has two mobile windows; sound enters through the oval window and exits via the round window [20, 32]. SSCD patients present with an abnormal “third mobile window” between the superior semicircular canal and the MCF, causing various vestibular and auditory symptoms that may require surgical intervention [4, 12, 15, 24, 26, 29, 34, 37]. In this study, we compared and analyzed preoperative variables and postoperative outcomes within a cohort of 350 consecutive SSCD MCF repairs performed over a decade at a single institution.

In this series, both unilateral (Cohort A) and bilateral (Cohort B) SSCD repair showed postoperative resolution of dizziness, with a lower resolution rate following bilateral repair, *p* = 0.0023. The unilateral repair cohort showed greater postoperative symptomatic resolution of autophony, amplification, hyperacusis, hearing, and dizziness than the bilateral repair cohort. Additionally, revisional surgery was associated with resolution of postoperative hearing, oscillopsia, autophony, and amplification. These finding align with previous studies [1, 8, 12, 15, 21, 30, 34] and emphasize the need for surgical intervention in cases where SSCD patients are experiencing persistent symptoms.

While bilateral disease has been associated with lower rates of symptomatic resolution postoperatively,[12, 15, 28, 34] few studies have compared the outcomes of unilateral vs. bilateral SSCD repair. We found that just over half (58%) of patients with bilateral disease underwent only unilateral repair in our cohort, which is consistent with prior reports [21]. These patients may have less severe disease or may experience sufficient symptomatic relief with unilateral repair and thus do not elect for a second-side surgery. These results suggest that unilateral repair may be effective for both unilateral and bilateral SSCD. This potential to minimize operative risk without a requirement for additional surgery is of particular importance for patients with substantial comorbidities or greater surgical risk. Future studies should investigate the predictive factors for unilateral repair in patients with bilateral SSCD.

SSCD has been shown to cause various auditory and vestibular symptoms [1, 5, 8, 12, 15, 19–22, 30, 34, 36]. In accordance with previous findings by Romiyo et al. [28] and Mozaffari et al.,[21] our study showed that tinnitus, dizziness, autophony, amplification, aural fullness, and disequilibrium as were the most prominent preoperative symptoms (Table 2). We did not note any significant differences in preoperative symptoms between patients undergoing unilateral and bilateral SSCD repair. As previous reports have shown bilateral SSCD to be associated with greater risk of preoperative autophony, disequilibrium, tinnitus, and dizziness [12, 15, 28, 34], additional investigation is necessary to identify predictive preoperative factors to distinguish between patients with bilateral SSCD requiring single- or second-side repair. For postoperative symptoms, auditory and vestibular symptom resolution following SSCD repair has been reported in previous studies [1, 8, 12, 15, 21, 28, 30, 34]. A case series of 156 repairs by Romiyo et al. reported postoperative symptomatic resolution in amplification, autophony, tinnitus, and headache [28]. Similarly, a larger series of 229 SSCD cases by Mozaffari et al. reported significantly higher postoperative symptomatic improvement in hyperacusis, hearing loss, dizziness, and disequilibrium in cases with unilateral repair compared to bilateral repair [21]. Similarly, a study by Crane et al. found that in patients with significant autophony symptoms, SSCD plugging improved symptoms in 94% of patients [8]. Findings of the aforementioned studies align with our findings of greater postoperative symptomatic resolution of autophony, amplification, hyperacusis, hearing, and dizziness in unilateral relative to the bilateral repair (Table 3). Regarding postoperative symptomatic resolution in unilateral versus bilateral SSCD repair, studies have reported bilateral SSCD cases presented less postoperative symptomatic resolution [12, 15, 28, 34]. Similarly, Chen et al. reported that unilateral SSCD patients presented greater postoperative symptom improvement in dizziness than bilateral SSCD patients, *p* = 0.0659 [12]. Overall, this suggests that though there are varying clinical symptoms in bilateral SSCD patients, there are still common clinical symptoms that are more prevalent and persistent compared with unilateral SSCD. The current findings and previous literature emphasize the importance of considering postoperative outcomes when choosing surgical intervention, as unilateral SSCD patients seemed to benefit more than bilateral SSCD patients. Additionally, given bilateral SSCD presents increased complexity for both presenting symptoms and postoperative symptomatic resolution compared to unilateral SSCD, patients that are candidate for bilateral repair should be informed of adverse outcomes.

Perioperative CSF leak is seen in 25.4% of the current cohort. Conditions associated with increased intracranial pressure, such as obesity or hypertension, increase the risk of spontaneous CSF leaks [16]. However, the method of intervention for SSCD repair is also critical, as CSF leak can be reduced by changing pressure differences within the ear. For example, the MCF approach allows the dehiscence to be seen and is, therefore, less invasive as it can be directly accessed [24]. On the other hand, the transmastoid approach limits dehiscence visibility, requires a drill on the canal, and the risk of potentially suctioning perilymph [24]. This risks more pressure damage to the neurons or surrounding hair cells and increases the chance for spontaneous CSF leaks [24]. Though the MCF approach gives a better view of the dehiscence and be beneficial during repair, it has a higher morbidity than the transmastoid route [16]. Therefore, endoscopic assistance is another approach that may improve the MCF technique [16, 22]. Plugging and capping techniques are also associated with higher success rates than resurfacing, and the combination of plugging and resurfacing achieves better long-term symptom control than resurfacing alone [10]. When only resurfacing is performed, a complete sealing of the dehiscence is not guaranteed, resulting in increased sensitivity to pressure changes and thus increased susceptibility to CSF leaks. [10]

History of ear anomaly was noted in 22.9% of the SSCD patients. There is a myriad of symptoms that present in SSCD that are also demonstrable in other otolaryngologic conditions. One such anomaly is mastoiditis, a condition where the mastoid bone in the inner ear cannot properly receive air from the eustachian tube (ET) [14]. Kawase et al. found that autophony might be significantly worse in patients with poorly developed mastoid cavity [14]. This is because sound transmission from the pharyngeal cavity to the middle ear through the ET is negatively affected due to a poorly-aerated mastoid [14]. Considering the overlap of symptoms between mastoiditis, other similar ear anomalies, and SSCD, repair of the dehiscence may offer resolution of debilitating afflictions.

This study is not without limitations. While all cases were performed with consistent operative techniques and all patients returned for their immediate follow-up, with careful clinical care documentation in their medical records, this study is retrospective and a single institution’s experience. Reporting bias is a limitation due to the subjective method of obtaining patients’ symptoms pre- and post-operatively. Patients may be experiencing a symptom but may be unable to effectively communicate it. For patients that obtained another surgery from a different institution, only the surgery done at our institution and its corresponding symptom data were reportable. Despite accounting for differences in repair operations, we did not control for laterality of SSCD. This may introduce bias, as differences in presentation and outcome have been noted between unilateral and bilateral SSCD [12, 15, 28, 34]. However, our findings of greater symptom resolution with unilateral relative to bilateral repair are consistent with prior studies [21, 35]. The present study presents the largest known single institution cohort of SSCD repairs. However, future studies should be performed to further investigate the role of SSCD laterality and to develop recommendations for counseling patients for unilateral or bilateral repair.

## Conclusion

SSCD is a rare disease, and the exact cause is unknown. Differences in outcomes between patients may have to do with bilateralism of SSCD. Treatment of SSCD is guided by symptom severity. For patients with mild to no symptoms, a conservative approach offers a non-invasive trigger avoidance strategy. However, for those with more debilitating symptoms surgical repair may offer relief. Our study findings suggest that surgical repair via the MCF can significantly resolve auditory, vestibular, and neurological symptoms of patients with SSCD. These findings may also apply to patients with SSCD and co-morbid ear anomalies. Although this is one of the largest single institution studies of SSCD to date, multi-institution populations would be beneficial to draw stronger conclusions.

## Data Availability

Not Applicable.

## Abbreviations

SSCD: Superior semicircular canal dehiscence
SSC: Superior semicircular canal
HRCT: High resolution computed tomography
cVEMP: Cervical Vestibular Evoked Myogenic Potentials
oVEMP: Ocular Vestibular Evoked Myogenic Potentials
MCF: Middle cranial fossa
CSF: Cerebrospinal fluid
ET: Eustachian tube

## References

[1] Office of the Surgeon General (US) (2004) Bone Health and Osteoporosis: A Report of the Surgeon General. Office of the Surgeon General (US). Accessed May 28, 2022. http://www.ncbi.nlm.nih.gov/books/NBK45513/20945569

[2] Arsenault JJ, Romiyo P, Miao T et al (2020) Thinning or dehiscence of bone in structures of the middle cranial fossa floor in superior semicircular canal dehiscence. J Clin Neurosci 74:104–108. 10.1016/j.jocn.2020.01.082 32044131 10.1016/j.jocn.2020.01.082

[3] Baxter M, McCorkle C, Trevino Guajardo C et al (2019) Clinical and physiologic predictors and postoperative outcomes of near dehiscence syndrome. Otol Neurotol 40(2):204–212. 10.1097/MAO.0000000000002077 30570606 10.1097/MAO.0000000000002077 PMC6326856

[4] Beckett JS, Chung LK, Lagman C et al (2017) A method of locating the dehiscence during middle fossa approach for superior semicircular canal dehiscence surgery. J Neurol Surg B Skull Base 78(4):353–358. 10.1055/s-0037-1601886 28725523 10.1055/s-0037-1601886 PMC5515662

[5] Bi WL, Brewster R, Poe D et al (2017) Superior semicircular canal dehiscence syndrome. J Neurosurg 127(6):1268–1276. 10.3171/2016.9.JNS16503 28084916 10.3171/2016.9.JNS16503

[6] Bigelow RT, Agrawal Y (2015) Vestibular involvement in cognition: visuospatial ability, attention, executive function, and memory. J Vestib Res 25(2):73–89. 10.3233/VES-150544 26410672 10.3233/VES-150544

[7] Chemtob RA, Noij KS, Qureshi AA, Klokker M, Nakajima HH, Lee DJ (2019) Superior canal dehiscence surgery outcomes following failed round window surgery. Otol Neurotol 40(4):535–542. 10.1097/MAO.0000000000002185 30870372 10.1097/MAO.0000000000002185

[8] Crane BT, Lin FR, Minor LB, Carey JP (2010) Improvement in autophony symptoms after superior canal dehiscence repair. Otol Neurotol 31(1):140–146. 10.1097/mao.0b013e3181bc39ab 20050268 10.1097/mao.0b013e3181bc39ab

[9] Crovetto M, Areitio E, Elexpuru J, Aguayo F (2008) Transmastoid approach for resurfacing of superior semicircular canal dehiscence. Auris Nasus Larynx 35(2):247–249. 10.1016/j.anl.2007.06.010 17900840 10.1016/j.anl.2007.06.010

[10] Fang Z, Tian R, Jia YT, Xu TT, Liu Y (2017) Treatment of cerebrospinal fluid leak after spine surgery. Chin J Traumatol 20(2):81–83. 10.1016/j.cjtee.2016.12.002 28336418 10.1016/j.cjtee.2016.12.002 PMC5392710

[11] Fiorino F, Barbieri F, Pizzini FB, Beltramello A (2010) A dehiscent superior semicircular canal may be plugged and resurfaced via the transmastoid route. Otol Neurotol 31(1):136–139. 10.1097/MAO.0b013e3181b76b9e 19707169 10.1097/MAO.0b013e3181b76b9e

[12] Jacky Chen CH, Nguyen T, Udawatta M et al (2019) Clinical assessment of patients with bilateral superior semicircular canal dehiscence. World Neurosurg 126:e1549–e1552. 10.1016/j.wneu.2019.03.205 30928582 10.1016/j.wneu.2019.03.205

[13] Kaur T, Johanis M, Miao T et al (2019) CT evaluation of normal bone thickness overlying the superior semicircular canal. J Clin Neurosci 66:128–132. 10.1016/j.jocn.2019.05.001 31103254 10.1016/j.jocn.2019.05.001

[14] Kawase T, Hori Y, Kikuchi T, Oshima T, Kobayashi T (2008) The effects of mastoid aeration on autophony in patients with patulous eustachian tube. Eur Arch Otorhinolaryngol 265(8):893–897. 10.1007/s00405-007-0560-1 18180937 10.1007/s00405-007-0560-1

[15] Lagman C, Ong V, Chung LK et al (2017) Pediatric superior semicircular canal dehiscence: illustrative case and systematic review. J Neurosurg Pediatr 20(2):196–203. 10.3171/2017.3.PEDS1734 28548616 10.3171/2017.3.PEDS1734

[16] Lobo BC, Baumanis MM, Nelson RF (2017) Surgical repair of spontaneous cerebrospinal fluid (CSF) leaks: A systematic review. Laryngoscope Investig Otolaryngol 2(5):215–224. 10.1002/lio2.75 29094066 10.1002/lio2.75 PMC5655559

[17] Mahendran S, Sunkaraneni VS, Baguley DM, Axon PR (2007) Superior semicircular canal dehiscence with a large tegmental defect. J Laryngol Otol 121(2):189–191. 10.1017/S0022215106004178 17059625 10.1017/S0022215106004178

[18] Mannion AF, Impellizzeri FM, Naal FD, Leunig M (2015) Women demonstrate more pain and worse function before THA but comparable results 12 months after surgery. Clin Orthop Relat Res 473(12):3849–3857. 10.1007/s11999-015-4479-3 26224293 10.1007/s11999-015-4479-3 PMC4626491

[19] Minor LB (2005) Clinical manifestations of superior semicircular canal dehiscence. Laryngoscope 115(10):1717–1727. 10.1097/01.mlg.0000178324.55729.b7 16222184 10.1097/01.mlg.0000178324.55729.b7

[20] Minor LB, Solomon D, Zinreich JS, Zee DS (1998) Sound- and/or pressure-induced vertigo due to bone dehiscence of the superior semicircular canal. Arch Otolaryngol Head Neck Surg 124(3):249. 10.1001/archotol.124.3.249 9525507 10.1001/archotol.124.3.249

[21] Mozaffari K, Willis SL, Unterberger A et al (2021) Superior semicircular canal dehiscence outcomes in a consecutive series of 229 surgical repairs with middle cranial fossa craniotomy. World Neurosurg 156:e229–e234. 10.1016/j.wneu.2021.09.038 34547526 10.1016/j.wneu.2021.09.038

[22] Nguyen T, Lagman C, Sheppard JP et al (2018) Middle cranial fossa approach for the repair of superior semicircular canal dehiscence is associated with greater symptom resolution compared to transmastoid approach. Acta Neurochir 160(6):1219–1224. 10.1007/s00701-017-3346-2 29022108 10.1007/s00701-017-3346-2

[23] Nguyen T, Sheppard JP, Duong C et al (2019) Age and gender considerations on the symptomology in patients with superior semicircular canal dehiscence: a systematic review and case illustration. J Clin Neurosci 65:112–120. 10.1016/j.jocn.2019.04.006 31078378 10.1016/j.jocn.2019.04.006

[24] Palma Diaz M, Cisneros Lesser JC, Vega AA (2017) Superior semicircular canal dehiscence syndrome - diagnosis and surgical management. Int Arch Otorhinolaryngol 21(2):195–198. 10.1055/s-0037-1599785 28382131 10.1055/s-0037-1599785 PMC5375705

[25] Pleym H, Spigset O, Kharasch ED, Dale O (2003) Gender differences in drug effects: implications for anesthesiologists. Acta Anaesthesiol Scand 47(3):241–259. 10.1034/j.1399-6576.2003.00036.x 12648189 10.1034/j.1399-6576.2003.00036.x

[26] Ramsey MJ, McKenna MJ, Barker FG (2004) Superior semicircular canal dehiscence syndrome: case report. J Neurosurg 100(1):123–124. 10.3171/jns.2004.100.1.0123 14743923 10.3171/jns.2004.100.1.0123

[27] Rodgers B, Lin J, Staecker H (2016) Transmastoid resurfacing versus middle fossa plugging for repair of superior canal dehiscence: comparison of techniques from a retrospective cohort. World J Otorhinolaryngol Head Neck Surg 2(3):161–167. 10.1016/j.wjorl.2016.11.001 29204562 10.1016/j.wjorl.2016.11.001 PMC5698534

[28] Romiyo P, Duong C, Ng E et al (2019) Superior semicircular canal dehiscence postoperative outcomes: A case series of 156 repairs. J Clin Neurosci 68:69–72. 10.1016/j.jocn.2019.07.053 31383473 10.1016/j.jocn.2019.07.053

[29] Trieu V, Pelargos PE, Spasic M et al (2017) Minimally invasive middle fossa keyhole craniectomy for repair of superior semicircular canal dehiscence. Oper Neurosurg 13(3):317–323. 10.1093/ons/opw046 10.1093/ons/opw046 28521355

[30] Ung N, Chung LK, Lagman C et al (2017) Outcomes of middle fossa craniotomy for the repair of superior semicircular canal dehiscence. J Clin Neurosci 43:103–107. 10.1016/j.jocn.2017.05.003 28622893 10.1016/j.jocn.2017.05.003

[31] Ward BK, van de Berg R, van Rompaey V et al (2021) Superior semicircular canal dehiscence syndrome: diagnostic criteria consensus document of the committee for the classification of vestibular disorders of the bárány society. J Vestib Res 31(3):131–141. 10.3233/VES-200004 33522990 10.3233/VES-200004 PMC9249274

[32] Ward BK, Carey JP, Minor LB (2017) Superior canal dehiscence syndrome: lessons from the first 20 years. Front Neurol 8:177. 10.3389/fneur.2017.00177 28503164 10.3389/fneur.2017.00177 PMC5408023

[33] Ward BK, Wenzel A, Ritzl EK et al (2013) Near-dehiscence: clinical findings in patients with thin bone over the superior semicircular canal. Otol Neurotol 34(8):1421–1428. 10.1097/MAO.0b013e318287efe6 23644303 10.1097/MAO.0b013e318287efe6 PMC3740012

[34] Wung V, Romiyo P, Ng E et al (2019) Sealing of superior semicircular canal dehiscence is associated with improved balance outcomes postoperatively versus plugging of the canal in middle fossa craniotomy repairs: a case series. J Neurosurg 133(2):462–466. 10.3171/2019.4.JNS19264 31252395 10.3171/2019.4.JNS19264

[35] Yang HH, Yang I, Gopen QS (2024) First-side and second-side repair of bilateral superior canal dehiscence. Laryngoscope 134(4):1882–1888. 10.1002/lary.31118 37937741 10.1002/lary.31118

[36] Yew A, Zarinkhou G, Spasic M, Trang A, Gopen Q, Yang I (2012) Characteristics and management of superior semicircular canal dehiscence. J Neurol Surg B Skull Base 73(6):365–370. 10.1055/s-0032-1324397 24294552 10.1055/s-0032-1324397 PMC3578588

[37] Ziylan F, Kinaci A, Beynon AJ, Kunst HPM (2017) A comparison of surgical treatments for superior semicircular canal dehiscence: a systematic review. Otol Neurotol 38(1):1–10. 10.1097/MAO.0000000000001277 27861193 10.1097/MAO.0000000000001277

